# More than Just Reacting: A Behavior Sequence Analysis of Victim–Offender Interactions During Violent Crime

**DOI:** 10.1177/08862605251355628

**Published:** 2025-07-29

**Authors:** Georgina Fuller, Louise E. Porter, Benoit Leclerc

**Affiliations:** 1 Griffith University, Brisbane, Australia

**Keywords:** violent offenders, sexual assault, domestic violence

## Abstract

The victim–offender interaction is an important part of violent incidents. However, very little research has examined how the actions of both parties’ shape and influence the behavior of the other. While the offender’s behavior and role in shaping the nature of the incident has received a large amount of attention, the corresponding influence of victim behavior is less understood. This study uses behavior sequence analysis to map victim–offender interactions during 149 cases of domestic violence, sexual assault, and physical assault (collectively referred to as physically assaultive crime) to understand how victim behavior shapes, and is shaped by, the offender during violent crime. By using the interpersonal circumplex—a probabilistic model of human behavior during social interactions—and its associated principle of complementarity to frame the analysis, the results emphasized the reciprocal influence a victim has on the offender. Results showed that while this dynamic mostly reflected other, non-violent, social interactions, key deviations in how victims responded to offenders may indicate that victims are not simply reacting to the offender but seeking their own ways to influence how the crime unfolds. These findings not only provide new insight into how physically assaultive crime unfolds but also have important implications for how we conceptualize a victim’s role and agency during violence.

## Introduction

Criminology has traditionally focused on the offender as the key to understanding and preventing crime. The victim’s role—that is, the process by which victims influence and shape the nature and outcome of a criminal incident—has been somewhat overlooked in comparison, remaining under researched and, therefore, poorly understood (Gittler, 1984; Fuller, 2015; Karmen, 2004). Failure to address this knowledge gap does more than limit our understanding of crime generally; it propagates negative, stereotypical conceptualizations around how victims behave and how crime unfolds.

Specifically, overlooking the victim ignores an integral component of crime—particularly violent crime. Routine Activity Theory, for example, identifies a suitable target or victim as one of the three essential elements that need to converge at the same time and space for crime (Cohen & Felson, 1979). Legally, it is often the presence, or absence, of a victim that determines whether a crime has taken place (Fuller, 2015). More broadly, the interaction that occurs between offender and victims during interpersonal violence means that both actors’ behaviors need to be examined to understand how a crime arrives at its conclusion. For example, Tedeschi and Felson (1994) emphasize the offender–victim interaction as part of their theory of coercive action. This social-interactionist perspective proposes that offenders are goal-oriented and engage in coercive actions with the purpose of producing change in the victim (p. 174). Similarly, Collins (2008) characterizes the offender–victim interaction as a process of “non-solidarity entrainment” (p. 147–148). During this mutual entrainment, both the offender and victim provide feedback that sustains the aggressive episode. Thus, while the offender may initiate, lead, or ultimately perpetrate the primary violence, this activity is not done in isolation from the victim—whose responses and actions likely also influence both the offender and the situation (Felson & Steadman, 1983; Luckenbill, 1980, 1982; Tedeschi & Felson, 1994).

Suggesting that the victim plays a role in shaping the crime does not mean that they bear any blame or responsibility for the nature and outcome of the incident. Instead, it characterizes the victim as an independent actor who is involved in the crime and who, despite their unwillingness, shapes and influences how it unfolds. It is the offender who chooses to commit the crime and who is therefore ultimately responsible for its outcome, regardless of how it manifests (i.e., completed or attempted; victim injured or not). Victims may make decisions that attempt to influence the outcome, and their actions may shape the nature of the criminal incident; however, this does not mean they should bear any of the responsibility.

The current study aims to explore the reciprocal influence that victims and offenders have on each other during violent crime. By using the interpersonal circumplex—a probabilistic model of human behavior during social interactions—to classify behavior and frame the victim/offender dynamic, this study will capture the varied and diverse ways victims behave during violence. It will also use behavior sequence analysis (Keatley, 2018) to map the interaction and gain further insight into the victim’s role from the initiation to the conclusion of a crime. Behavior sequence analysis is an emerging method that breaks an interaction down into a sequence of actions to facilitate greater understanding of the interpersonal dynamics. By adopting this approach, this study will begin to address the knowledge gap around how victims shape, influence, and impact the offender and, through them, how the crime unfolds.

### Crime as a Reciprocal Interaction Between Victims and Offenders

An interaction occurs when “the behaviors of one actor are consciously reorganised by, and influence the behavior of, another actor and vice versa” (Turner, 1988, p.13). Key to this definition is reciprocity and the way the thoughts, decisions, actions, movements, and behaviors of each party involved in the interaction are influenced and shaped by those of the other. This reciprocity can be seen in the basic sequence of events present in any interaction, human or non-human, which Mead (1934) termed the “triadic matrix.” Party A transmits signals into the environment through their movements or behavior. Party B perceives these signals and alters their own behavior in response, thereby creating and transmitting signals of their own. Party A then perceives B’s signals and responds by altering their own behavior and so on until the interaction ends (Turner, 1988). Human social interactions follow similar but slightly more complex patterns to non-human ones because of our ability to interpret, project, and predict others’ future behaviors based on their current actions and gestures; our sense of self and nuanced understanding of the impact of our own behaviors; and the influence of self-evaluation and reasoning on our decision-making process (see Turner, 1988). This complexity can be seen in Baldwin’s (1992, 1995) notions of relational schemas, which refer to an individual’s cognitive structures used to frame social interactions. These are derived from repeated experience and comprise inter-related images of self and other as well as interpersonal scripts that are used to interpret the social situation and behaviors of others. Functioning like a series of “if-then contingencies,” Baldwin states that interpersonal scripts contain expectations about the actor’s own goals and those of the other as well as the other person’s internal state, including their thoughts, feelings, and likely responses (Baldwin, 1992, 1995: 548).

Characterizing a criminal event as a particularly extreme and negative social interaction is not a new idea. Von Hentig, for example, described the roles of the victim and offender as complementary (in Kirchhoff (1994)) while Luckenbill (1977) characterized homicide as a “situated transaction” where fatal outcomes are shaped by the offender’s interpretation of the victim’s initial behavior and subsequent reactions. van Dijk (1994) described a rational-interactionist model of crime where “offenders are seen as the consumers of criminal gains and victims as the reluctant suppliers of criminal opportunities” (p. 105). More recently, victim–offender interactions during sexual assault and child sexual assault have been described as dysfunctional, maladapted versions of those in more conventional, non-abusive relationships (Alison & Stein, 2001; Bennell et al., 2001). Similarly, research into offender crime scripts has also highlighted the interactive nature of violent crime (Boxall et al., 2018; Leclerc et al., 2014; Copes et al., 2012; Smith, 2016). For example, in their examination of child sexual abuse crime scripts, Leclerc et al. (2014) found that victim response impacted the nature and outcome of the abuse. They found that victim compliance predicted the intrusiveness of the offender and likelihood of the victim engaging in sexual behaviors. They also found that victim compliance was more likely when the offender used persuasive actions—such as showing affection, giving bribes or drugs and alcohol, or using, or threatening to use, force—as opposed to non-persuasive strategies such as acting on the spur of the moment (Leclerc et al., 2014). Yet despite these studies, much research has continued to focus on offender behavior during crime (and its impact on victims) and overlook the reciprocal influence of victim behavior and its impact on the offender.

Viewing crime as an interaction allows us to reconceptualize and re-examine victims’ role from a perspective that emphasizes their agency during the incident. Victim agency during crime has received very little attention and refers to the extent to which victim behavior reflects conscious, deliberate, and intentional attempts to influence the crime in reference to some underlying, self-motivated goal. This definition is derived from the more general concept of agency within Bandura’s social cognitive theory (SCT). SCT proposes that humans are agents who actively shape, and are shaped by, the environment in which they function. When acting with agency, humans’ behavior will reflect their underlying motivation and be influenced by intrapersonal, behavioral, and environmental characteristics (see Bandura 1999, 2006). Adopting an agentic perspective acknowledges the victim as an actor during crime with their own goals, motivations, and decision-making processes distinct from, but influenced by, those of the offender—and vice versa. Viewing crime as an interaction also enables us to apply our understanding of how humans behave in social interactions generally to provide valuable insight into victim and offender behavior during crime.

### The Interpersonal Circumplex: A Model of Human Behavior During Interactions

The interpersonal circumplex (IPC) is a probabilistic model of human behavior during social interactions (Plutchik & Conte, 1997; Wiggins, 1996). It classifies behavior according to two bisecting axes: one that represents power (dominance versus submission) and affiliation (hostility vs. cooperation) (Leary, 1957; Kiesler, 1983; Wiggins, 1996). An action’s position relative to both axes signifies the behavioral styles it aligns with. On the vertical power axis, dominance reflects how an individual expresses control during an interaction, while submission reflects the yielding of it (Leary, 1957; Wiggins, 1996). Fuller et al. (2023) have recently used the IPC to classify victim behaviors that occurred during three types of violent crime: domestic violence, sexual assault, and physical assault. Victim actions found to reflect a dominant behavioral style include defending themselves or evading the offender, leaving the scene, talking to the offender, and refusing to cooperate. Submissive victim behaviors include cowering, engaging in sex acts with the offender, saying no, doing nothing, and uncontrollable responses such as freezing (Fuller et al., 2023). On the horizontal affiliation axis, hostility is defined as opposing and/or antagonistic behaviors (Leary, 1957; Wiggins, 1996), with victim actions such as arguing, screaming, calling for help, confronting, or fighting the offender found to reflect a hostile behavioral style (Fuller et al., 2023). Alternatively, cooperation comprises behaviors that invite collaboration and/or support (Leary, 1957; Wiggins, 1996) and comprises victim behaviors such as cooperating with the offender and negotiating/compromising (Fuller et al., 2023).

In addition to classification, the IPC also presents hypotheses about the likely reactions to these behaviors through the principle of complementarity. This principle proposes that an individual’s behavior during an interaction elicits an increased likelihood of a specific, complementary, response (Kiesler, 1983). It manifests differently on each axis as reciprocity on the dominance/submission axis (i.e., dominant behavior is likely to elicit submissive response, and vice versa) and correspondence on the hostility/co-operation axis (i.e., co-operative behavior is likely to elicit a co-operative response while hostility is likely to elicit a hostile response). The principle of complementarity is probabilistic not deterministic, meaning that it reflects the likelihood of a particular response based on a specific type of behavior (Keisler, 1983). The IPC, therefore, provides a basis for describing and understanding victim behavior as it relates to, shapes, and is shaped by offender behavior.

The body of research that has quantitatively modeled the IPC during violent crime has identified the principle of complementarity operating in offender/victim interactions. For example, Bennell et al. (2001), modeled behavior between adult offenders and child victims during child sexual assault. They included 19 behaviors in total, though only four were victim behaviors. This included “victim masturbates the offender” and “victim performs oral sex on the offender” as well as an umbrella category- “victim participates.” Bennell et al.’s study showed that most victim behaviors were correlated with the corresponding offender behaviors modeled by the principle of complementarity. However, the limited descriptions of these behaviors provide little insight into the interaction from a victim-led perspective. Porter and Alison (2006) used smallest space analysis to explore the victim and offender behavior during robberies involving multiple offenders. Despite only including a small number of victim behaviors, they found support for the principle of complementarity with cooperative offender behaviors likely to result in similarly compliant victim behaviors. Additionally, victim behaviors such as struggling, refusing to acquiesce to the offender, and running away did not correlate with dominant, hostile, or cooperative offender behavior. As such, they concluded that these behaviors likely reflected dominant victim behavior and would have correlated with submissive offender behaviors, had any been identified and included. Finally, Porter and Alison (2004) found some support for the principle of complementarity in the relationship between victim and offender behavior in group rape, although they did not test the full range of victim behaviors. They found that aggressive or violent offender behavior on the hostility-co-operation axis was correlated with victim behavior such as screaming or physically fighting and that victims pleading with the offender and crying were highly correlated with compliance-gaining offender behaviors such as threatening the victim not to scream or report, threatening the victim to comply, and letting the victim go. They concluded that some victims may be attempting their own compliance-gaining strategies on the offenders, but this was not directly tested. Similarly, pseudo-submissive behaviors from the offender, such as a confidence approach (gaining victim trust) and demeaning or insulting the victim, were highly correlated with victim responses such as struggling, refusing to comply, and running away. The authors suggest that this indicates victims are adopting a dominant behavioral style in reaction to submissive offender behavior. The study, therefore, suggests that victim behavior not only reflects the domains and principles of the IPC, but that victims may have some agency in their behavioral style with respect to asserting their own goals (of crime avoidance).

Outside of these studies, research into crime completion and victim resistance has also shown how victim behavior influences the offender and outcome of the crime. While these studies do not use the IPC, their results can be interpreted via the principle of complementarity. Failing to complete a crime, for example, can be viewed as a form of offender submission, meaning that any victim behavior correlated with this outcome may reflect a dominant behavioral style. Studies have found that crimes are less likely to be completed in incidents when the victim resists the offender (Block & Skogan, 1986; Fisher et al., 2007; Guerette & Santana, 2010; Leclerc & Wortley, 2014; Siegal et al., 1989; Ullman, 1997). Block and Skogan (1986), for example, examined the impact of victim resistance on robbery and rape. They found that resistance was negatively correlated with crime completion, meaning that greater victim resistance resulted in fewer completed robberies and rapes. Robbery victims who did not resist or engage in any self-protective behavior were more likely to have property stolen compared with those who did (Block & Skogan, 1986). Similarly, Tark and Kleck (2004) found that resistance including struggling, yelling, and attacking the offender decreased the likelihood of property loss during robberies, burglaries, and thefts. Though victim resistance often included a range of behaviors reflecting both dominant and hostile behavioral styles, these studies included victim behaviors such as resisting or not cooperating with the offender which Fuller et al. (2023) found to reflect a dominant behavioral style.

At the same time, victim resistance is also correlated with increased likelihood of victim injury, highlighting how this type of behavior can result in offenders being more hostile and aggressive to victims. According to the principle of complementarity, this hostile offender behavior is more likely in response to hostile victim behavior, and vice versa. Bachman and Carmody (1994) examined the likelihood of victim injury in 656 cases of domestic violence from the National Crime Victimization Survey in the United States. This likelihood doubled during incidents of domestic violence in which the victim resisted compared with those where they did not (Bachman & Carmody, 1994). Later research supported this, finding positive correlations between resistance behaviors such as wrestling, fighting, striking, or using a weapon and victim injury (Bachman et al., 2002). Conversely, Powers and Simpson (2012) found negative correlations between victim injury during domestic violence and compliance-gaining victim behaviors such as pleading, crying, and placating the offender. The authors suggested that specific forms of victim behavior may be related to particular responses from the offender and vice versa. Similarly, Fisher et al. (2007) examined multiple forms of sexual assault and found a relationship between victim and offender behavior. Forceful, physical resistance (i.e. attacking the offender) was more common in incidents of sexual assault where the offender was physically forceful toward the victim such as during rape or unwanted sexual contact with force or threats of force. Alternatively, non-forceful physical and verbal resistance such as avoiding the offender, running away, removing the offender’s hands, negotiating with the offender, or telling/pleading/begging the offender to stop, were more common in incidents where the offender was non-forceful with the victim. These included incidents of sexual coercion where the offender used non-physical threats, promises of rewards, or otherwise verbally pestered or pressured the victim. Fisher et al. (2007) concluded victims chose the type of resistance based on its perceived efficacy within the current situation as well as parity with the offender’s actions—which is in line with the principle of complementarity. Further, Fuller et al.’s (2023) study showed fighting, striking, and wrestling with the offender reflected a hostile victim behavioral style, and thus would increase the likelihood of aggression and hostility from the offender. Pleading and placating are forms of negotiation that reflected a cooperative behavioral style which, according to the IPC, would decrease the likelihood aggression or hostility in response.

Collectively, the results from both IPC- and non-IPC-focused research suggest that the victim–offender interaction during violent crime should follow the principle of complementarity. This, therefore, provides a useful theoretical foundation from which to explore the reciprocal influence the victim and offender have on each other during violence. It is hypothesized that victim behavior will be influenced by that of the offender. Specifically, it is hypothesized that, as per the principle of complementarity, offender behavior will more likely be followed by:

- victim submission when it aligns with a dominant behavioral style;
- victim dominance when it aligns with a submissive behavioral style;
- victim hostility when it aligns with a hostile behavioral style; and,
- victim cooperation when it aligns with a cooperative behavioral style.

However, as mentioned above, it is also hypothesized that this influence will be reciprocal with the victim’s behavioral style influencing the offender such that victim behavior will more likely be followed by:

- offender submission when it aligns with a dominant behavioral style;
- offender dominance when it aligns with a submissive behavioral style;
- offender hostility when it aligns with a hostile behavioral style; and,
- offender cooperation when it aligns with a cooperative behavioral style.

## Method

### Sample

This study utilized data from the Australian Institute of Criminology’s Database of Victimization Experiences (DoVE). The DoVE is a qualitative database of 730 psychological evaluations of victims who experienced domestic violence, sexual assault, physical assault, and robbery and subsequently sought compensation from Victim Services, New South Wales between 2005 and 2010. These psychological evaluations contain, among other things, victim narratives of their experience and provide information on the victim and offender interaction (see Fuller (2015) for more information on the DoVE including sample and methodology).

The current sample comprised 149 cases of domestic violence, sexual assault, and physical assault (see Table 1). Experiences of robbery were excluded because of the differences between this crime and the other three. Robbery commonly involved more instrumental violence as opposed to expressive or hostile forms present in domestic violence, sexual assault, and physical assault. Instrumental violence occurs when an offender’s goal is to obtain something—such as money, property, or status—possessed by someone else. In contrast, expressive violence occurs when the offender’s primary purpose is to harm or otherwise injure the victim (Youngs et al., 2016: 403). This distinction may lead to fundamental differences in victim experiences that would impact the consistency of the sample. In comparison, domestic violence, sexual assault, and physical assault share similarities in the nature of violence, which makes victim’s experiences more comparable to each other. However, these crime types also differ in specific ways, which provide additional scope to support the aims of this research. For example, physical assault involves non-sexual violence against the victim, sexual assault involves sexual violence, while domestic violence can include elements of both. Thus, including all three crimes provided greater scope to explore the victim–offender interactions across a range of violent situations.

**Table 1.** Sample Characteristics.

	Domestic Violence	Sexual Assault	Physical Assault	Total
Victim gender				
Female	23 (72%)	14 (70%)	37 (38%)	74 (49%)
Male	9 (28%)	6 (30%)	60 (62%)	75 (51%)
Victim age				
16–19	3 (9%)	4 (20%)	7 (7%)	14 (9%)
20–29	8 (25%)	2 (10%)	16 (16%)	26 (17%)
30–39	13 (41%)	6 (30%)	22 (23%)	41 (28%)
40–49	0 (0%)	2 (10%)	15 (15%)	17 (11%)
50–59	4 (13%)	1 (10%)	17 (17%)	22 (15%)
60 years or older	1 (3%)	1 (10%)	4 (4%)	6 (6%)
Not stated	3 (9%)	4 (20%)	16 (16%)	23 (15%)
Total	32	20	97	149

The 149 cases represented a subsample of DoVE cases of physically assaultive crime that comprised the experiences of individuals who were aged 16 years or older at the time of the crime and who provided enough detail about discrete incidents to be able to map the victim–offender interaction via behavior sequence analysis. This included clearly attributing actions, and the order in which they occurred, to the victim, offender, or third parties.

The decision to only include victims who were aged 16 years or older at the time of the assault was based on research showing age-related variation in children’s ability to accurately children’s capability to accurately recall their experiences. Specifically, being older at the time of trauma, as well as the severity of the experience, has been found to increase the accuracy of recall at later times (see Goodman et al., 2019 for a discussion). Excluding the experiences of children under the age of 16 years aimed to ensure the consistency in victim recollection. It was not possible to control for, or test, the accuracy of statements in cases where time had elapsed between the crime and victims’ engagements with Victim Services, New South Wales. However, as these statements were collected as part of official compensation claims, all cases were treated as accurate records of the incident.

Table 1 provides a summary of sample characteristics. Just half of the sample were male, though this varied by crime type. The majority of victims were aged between 30 and 39 at the time of their evaluation. It is worth noting that a small number of victim–offender pairings were involved in more than one incident. Specifically, 16 incidents of domestic violence involved one of six different victim–offender pairings; however, no victim–offender pairing appeared more than three times. While all these cases were included, the limitations of this approach are noted in the discussion section.

### Behavior Sequence Analysis

Behavior sequence analysis is based on probability theory and can be used to understand and map the progression of a social interaction (Beune et al., 2011; Keatley, 2018; Taylor et al., 2008). By breaking an incident into discrete behavioral chains, behavior sequence analysis enables researchers to quantitatively determine if a particular action is more likely to produce a specific response as opposed to any other behavior, as would be expected by chance (Keatley, 2018). This makes it highly suited to understanding how victims and offenders shape each other’s behavior during physically assaultive crime.

This research followed Keatley’s (2018) method for behavior sequence analysis. This involved three steps; *parsing* the incident into discrete behaviors, *categorizing* behaviors into similar groups, and *analyzing* the frequency by which one behavior (the antecedent) is followed by another (the sequitur). The same method has been used in prior research to map deceptive behavior during criminal interviews (Marono et al., 2018), understand the context around false confessions (Keatley et al., 2018), and explore non-victim perceptions of how sexual assault unfolds (Ellis et al., 2017).

### Parsing

Initially, each incident was broken down to create 149 discrete behavior sequences that mapped the interaction between the victim, offender, and any third parties. This process was conducted manually using Microsoft Excel. While a sequence could begin with either victim or offender behavior, the start of an incident was determined as the moment when the victim became aware of the offender, regardless of whether they realized the offender was a threat. This is in line with Mead’s (1934) triadic matrix as an individual is only able to shape, and be shaped by, the behavior of another if they are aware of their presence. The end of an incident was determined as the moment when the victim or offender were no longer in each other’s vicinity and able to interact. In cases where this moment was not described, the incident was considered to have concluded when the victim’s description ceased. All behaviors that occurred between an incident’s start and finish, including those by third parties and those that did not directly involve interaction (such as opening a door, or turning on a tap), were parsed. Sequence lengths varied substantially between incidents, ranging from two cases that contained only two behaviors (one victim and one offender action) to one that spanned 58 behaviors. On average, a sequence contained 11 behaviors in total. In 14% (*n* = 21) of cases, sequences comprised less than five behaviors, while those spanning over 30 behaviors comprised only 3% (*n* = 4).

### Categorization

The next step involved categorizing the individual behaviors according to the IPC’s behavioral styles. The victim’s behaviors were classified in line with Fuller at al. (2023) and reflected the discrete behaviors described by the victim in each case. For example, if victims described hitting, punching, kicking, or otherwise fighting the offender, these individual behaviors were classified as hostile. If they described crying, freezing, saying no, or otherwise not reacting, these behaviors were classified as submissive (see Table 2 for examples of how victim behavior aligned with the IPC according to Fuller et al. 2023). Fuller et al. (2023) noted that, in reality, actions would likely reflect elements of both axes; for example, dominance and cooperation or submission and hostility. As such, its classification here is intended to capture the behavioral style the action most strongly aligns with.

**Table 2.** Example of Victim and Offender Behaviors Aligned with IPC Behavioral Style.

IPC Behavioral Style	Example Victim Behavior (based on Fuller et al. 2023)	Example Offender Behavior (based on Alison and Stein (2001), Bennell et al. (2001), Hauffe and Porter (2009), Porter and Alison (2004), Porter and Alison (2006), Woodhams et al. (2020), Woods and Porter (2008))
Dominance	Defend self or other Evade the offender Leave the situation Talk to the offender Uncooperative behavior	Approach the victim Leave the situation Refuse victim request Force entry to property Steal victim property Detain or trap victim (incl. by binding, gagging, or drugging)
Submission	Cower Do nothing/not resist Say no Engage in sex acts with offender Uncontrollable responses (incl. freezing or tonic immobility)	Apologize to victim Ignore victim Cease or stop attack Drop weapon (either willingly or unwillingly) Let victim go
Cooperation	Cooperate with offender Negotiate with offender	Issue instruction or request to victim Threaten or make demand of victim Talk to victim (incl. asking questions, making inquiries about comfort etc.) Take victim to victim’s home
Hostility	Argue with offender Confront offender Fight offender Seek help Scream	Punch, hit, kick, stab, or otherwise physically assault victim Rape victim Insult, demean, degrade victim

For the offender behavior coding framework, offender-focused IPC classifications from seven previous studies were compared to create a new integrated coding framework. This approach was used by Hauffe and Porter (2009) and Woods and Porter (2008) in their studies of offender behavior. Specifically, the allocations of offender behaviors to IPC behavioral styles were compared across Porter and Alison (2004), Porter and Alison (2006), Bennell et al. (2001), Alison and Stein’s (2001), Woodhams et al. (2020), Woods and Porter (2008), and Hauffe and Porter (2009). While the studies varied in the terminology used, they all reflected the core four behavioral styles of dominance, submission, hostility, and cooperation. Where differences of classification occurred—for example, where one study classified an offender behavior as dominant while another classified it as hostile—behaviors were allocated based on majority or, where that was not possible, preference was given to studies whose classifications were based on quantitative results. Table 2 provides examples of offender behaviors aligned IPC behavioral styles. Importantly, the two classification frameworks do not mirror each other; that is, the same action may align with a different behavioral style depending on if it was performed by the victim or the offender. An example is talking. For offenders, this behavior aligns with a cooperative behavioral style while, for victims, it aligns with a dominant behavioral style. This is due to the different motivations and intentions behind the actions and reflects how differently they express power and affiliation (see Fuller et al. 2023 for a discussion).

To ensure that the victim, offender, or third part remained identifiable during analysis, the actor was identified using a letter (V, O, or 3P). The action’s behavioral style was similarly categorized using a corresponding letter (D, S, C, or H). Thus, for example, a victim crying was coded as V-S, while an offender kicking the victim was coded as O-H. Non-interactive actions, such as turning on a tap, were coded as NI. Initially, 942 offender behaviors were coded across the full sample and 543 victim behaviors.

However, multiple sequences included cases where an actor would engage in a series of different actions that all belonged to the same behavioral style. For example, an offender may yell, punch, and then kick a victim—all in succession. Specifically, there were 355 incidents where victim or offender behavior was followed by an action by the same actor, belonging to the same behavioral style. As this research focused on the movement from offender to victim behavior, and vice versa, these instances were combined into one action; for example, a sub-sequence that was initially coded as “O-H → O-H → V-H” became “O-H → V-H.” Additionally, there were 111 instances in which offender dominance was followed by offender hostility or vice versa, more than any other combination of behavioral style. To capture this unique pattern of offender behavior and understand its influence on victim behavior, an additional offender behavioral category of dominance/hostility (O-DH) was created, and these sub-sequences were condensed into this one coded action. Other combinations, such as OD → OC, did not occur to the same extent and so were not condensed. This process of condensing the sequences reduced the number of behaviors sequenced by 28%, with the final number presented in Table 3.

**Table 3.** Victim and Offender Behaviors, by IPC Behavioral Style.

Actor	IPC Behavior	*N*	% Grand Total	Frequency in Cases (*N* = 149)
Offender	Dominance	110	10.3	65
	Submission	25	2.3	17
	Hostility	224	21.1	127
	Cooperation	154	14.5	78
	Dominant/hostile	85	8.0	66
	Not interactive	2	0.2	2
	*Total offender*	*600*	*56.5*	
Victim	Dominance	184	17.3	108
	Submission	124	11.7	84
	Hostility	96	9.0	63
	Cooperation	42	4.0	32
	Not interactive	16	1.5	13
	*Total victim*	*462*	*43.5*	
Total behaviors sequenced		1,062	na	na

### Analysis

Finally, as per Keatley (2018), the sequences were analyzed as a collective. The frequency by which one behavior (the “antecedent” was followed by another (the “sequitur”) was mapped via a transition frequency matrix (TFM). A TFM was created for each antecedent/sequitur scenario of interest; specifically, the number of times an offender’s behavior is followed by a particular type of victim behavior and vice versa (see Tables 4 and 5). Despite being parsed, non-interactive behaviors and those of third parties were not included in the final analysis to preserve the focus on the interaction between victims and offenders. These behaviors were not removed from the sequence; rather, only offender or victim behaviors that were followed by an interpersonal offender or victim behavior were included in the TFM (see Tables 4 and 5).

**Table 4.** Adjusted Residuals, Offender Antecedent—Victim Sequitur.

			Sequitur (Victim)			
			V-D	V-S	V-H	V-C
Antecedent (Offender)	O-D	Count	***22***	6	6	1
		Expected count	***14.6***	9.9	7.2	3.3
		Adjusted residual	***2.7***	–1.5	–.5	–1.4
	O-S	Count	4	1	1	1
		Expected count	2.9	2.0	1.4	.7
		Adjusted residual	.8	–.8	–.4	.4
	O-H	Count	66	50	31	***5***
		Expected count	63.3	43.0	31.4	***14.3***
		Adjusted residual	.6	1.7	–.1	***–3.5***
	O-C	Count	***25***	***16***	20	***23***
		Expected count	***35.0***	***23.7***	17.4	***7.9***
		Adjusted residual	***–2.6***	***–2.2***	.8	***6.5***
	O-DH	Count	20	***20***	10	***1***
		Expected count	21.2	***14.4***	10.5	***4.8***
		Adjusted residual	–.4	***1.9***	–.2	***–2.0***

*Note.* Does not include cases where an offender behavior is followed by other offender behavioral styles, V-NI, or third-party actions. Significant findings represented in bold italics. O-D = offender dominance; O-S = offender submission; O-H = offender hostility; OC = offender cooperation; O-DH = offender dominance/hostility; V-D = victim dominance; V-S = victim submission; V-H = victim hostility; V-C = victim cooperation.

**Table 5.** Adjusted Residuals, Victim Antecedent—Offender Sequitur.

			Sequitur (offender)				
			O-D	O-S	O-H	O-C	O-HD
Antecedent (victim)	V-D	Count	***13***	8	***78***	21	18
		Expected count	***22.9***	8.0	***67.4***	20.6	19.2
		Adjusted residual	***−3.1***	.0	***2.5***	.1	*−*.4
	V-S	Count	***17***	3	27	9	7
		Expected count	***10.5***	3.6	30.8	9.4	8.8
		Adjusted residual	***2.5***	***−***.4	***−*** 1.1	***−***.2	***−***.7
	V-H	Count	9	4	32	***1***	***14***
		Expected count	10.0	3.5	29.3	***8.9***	***8.3***
		Adjusted residual	***−***.4	.3	.8	***−3.2***	***2.4***
	V-C	Count	***10***	2	***7***	***13***	2
		Expected count	***5.6***	2.0	***16.6***	***5.1***	4.7
		Adjusted residual	***2.1***	.0	***−3.5***	***4.1***	***−*** 1.4

*Note.* Does not include cases where a victim behavior is followed by other victim behavioral styles, O-NI, or third-party actions. Significant findings represented in bold italics. O-D = offender dominance; O-S = offender submission; O-H = offender hostility; OC = offender cooperation; O-DH = offender dominance/hostility; V-D = victim dominance; V-S = victim submission; V-H = victim hostility; V-C = victim cooperation.

Data analysis was conducted using SPSS version 27. The Fisher–Freeman–Halton test, a variation of Fisher’s exact test, was used in place of a chi-square to determine whether the antecedent behavioral style was significantly more likely to be followed by a specific sequitur behavioral style then expected by chance. Like Fisher’s exact, the Fisher–Freeman–Halton test is suited to determining significance in cases where contingency tables contain cells with expected counts less than five. However, unlike Fisher’s exact, it can be applied to contingency tables greater than 2x2, such as those in this study. Due to sample size, adjusted standardized residuals were used to determine the significance of specific antecedent-sequitur pairings. Residuals reflect the difference between observed and expected cell counts (Delucchi, 1993). The standard rule-of-thumb that cells with residuals greater than ±1.96 are significantly larger than expected was used to identify significant relationships between victim behavior and offender response, and vice versa.

## Results

### Offender Cue-Victim Response Patterns

The Fisher–Freeman–Halton exact test showed that there was a significant association between antecedent offender behavioral styles and the victim behavioral styles that immediately followed (*p* < .01). Table 4 shows the adjusted residuals for the TFM, with significant associations in bold. The results partially align with the principle of complementarity. On the affiliation (cooperation—hostility) axes, offender cooperation was more frequently followed by corresponding victim cooperation than expected by chance. At the opposite end, however, offender hostility did not have a significant association with corresponding victim hostility, as would be expected according to the principle of complementarity. However, it was less frequently followed by victim cooperation than was expected by chance. This is in line with the principle of complementarity as it is expected that offender hostility would be unlikely to invite victim cooperation in response. Finally, negative associations were found between offender cooperation and victim dominance and victim submission. This was unexpected as these reflect responses between axes as opposed to within—something not modeled by the IPC.

In terms of the impact of offender dominance or submission, offender submission had no significant association with any victim behavioral style—though there were only seven instances of submissive offender behavior across the full sample. It is possible that this reflects a bias in the sample as, in all cases, crimes were completed. Had the sample contained attempted crimes, like those examined by Block & Skogan (1986) and Tark & Kleck (2004), more instances of offender submission may have been identified.

Offender dominance without hostility was more commonly followed by victim dominance than expected by chance. This was opposite to expected according to the IPC principle of complementarity. There was, however, a significant association between offender dominance with hostility and victim submission with submissive victim behavior more common following this style of offender behavior than expected by chance. This is in line with the principle of complementarity and may reflect the unique circumstances of violent crime.

### Victim Cue-Offender Response Patterns

Inverting the perspective, the Fisher–Freeman–Halton exact test showed that there was an association between specific victim behavioral styles and the offender behavioral styles that succeeded it (*p* < .01). Table 5 shows that, in line with the principle of complementarity, victim cooperation was more commonly followed by offender cooperation and less commonly followed by offender hostility. Similarly, when victims displayed a hostile behavioral style, this was more commonly followed by offender dominance/hostility and less commonly followed by offender cooperation. On the power (dominance–submission) axis, victim dominance had a negative association with offender dominance, but it was not followed by offender submission, as expected. Victim submission, however, was positively associated with offender dominance, as expected by the principle of complementarity.

Two significant results were contrary to the relationships modeled by the principle of complementarity as they reflected interactions between axes as opposed to within. First, that victim cooperation was found to be more commonly followed by offender dominance, and second, that victim dominance was more commonly followed by offender hostility. The IPC does not provide insight into cue-response between axes. It is worth nothing however that, due to the model’s bisecting structure, a single action would reflect characteristics of both axes, for example, dominance and cooperation or submission and hostility.

## Discussion

This study aimed to explore the reciprocal influence victims and offenders have on each other during violent crime. Overall, the results showed that victim behavior both influences, and is influenced by, that of the offender in ways that align with the IPC’s principle of complementarity. Such a finding highlights the importance of acknowledging and understanding the varied and nuanced ways victims shape and influence a violent offender and situation.

Using sequence analysis highlighted the influence of different victim behavioral styles on the offender. Classifying victim and offender behavior to reflect the four styles of the IPC—dominance, submission, hostility, and cooperation—provided greater insight into the dynamic between victim and offender. For example, cooperative and hostile behavioral styles are opposite manifestations of affiliation and reflect the ways parties build, maintain, or deny connection during an interaction (Leary, 1957; Wiggins, 1996). Results showed that cooperative victim and offender behaviors were more commonly met with cooperative responses than expected by chance, while hostile behaviors less commonly received cooperation in response. Cooperative victim behaviors, such as complying or negotiating with the offender, were also less commonly met with hostile offender responses such as hitting, kicking, or otherwise behaving aggressively toward the victim. However, when victims behaved in a similarly hostile manner, offenders more often responded with aggressive, dominant behaviors. Such findings are in line with the IPC and the principle of complementarity. In the current context, these findings regarding cooperative and hostile offender and victim behavioral styles demonstrate the range of ways victims may gain, or eschew, compliance from the offender and vice versa.

On the other hand, dominant and submissive behavioral styles reflect the degree to which interacting parties express or yield control (Leary, 1957; Wiggins, 1996). According to the principle of complementarity, these styles have a reciprocal relationship, with dominant behaviors more like to be met with submission and vice versa. This was reflected in the ways offenders responded to victims during physically assaultive crimes. For example, while the frequency of submissive offender behaviors was likely too low to be meaningfully analyzed, offenders were less likely to respond dominantly when victims behaved dominantly—for example, by defending themselves, not cooperating, leaving, talking to, or evading the offender. Similarly, when victims behaved submissively by cowering, engaging in sex acts, doing nothing, saying no, or having an uncontrollable response, offenders were more likely to respond dominantly.

However, these patterns deviated from what was hypothesized in the ways victims responded to the offender. It was hypothesized that offender dominance would be more commonly followed by victim submission, yet the results indicated that it was more commonly followed by victim dominance than expected. One reason for this finding may relate to the circumstance of a violent interaction. Dominant offender behavior included arriving, locking the door, breaking in, refusing to leave, or refusing to follow victim direction, and these behaviors may be more likely to occur at the start of an interaction when the victim is unaware of the level of threat or danger the offender poses. At this time, a victim may behave dominantly—such as by trying to leave or asking questions—to try to control the offender or situation. However, once they become aware of the level of danger, they change their behavioral style and submit. While this study did not examine where in the sequence these sub-interactions occurred, this would also explain why victims were significantly more likely to respond with submission when offenders displayed a mixture of dominance and hostility. This highly aggressive, highly controlling string of behavior would likely leave the victim in no doubt as to the offender’s intent and level of danger resulting in a greater likelihood of submission.

The unique context of a violent, as opposed to non-violent, interaction may also explain the unexpected findings that reflected responses between the power and affiliation axes as opposed to those previously discussed that occurred within. While offender cooperation was positively associated with victim cooperation in response, it was also negatively associated with victim dominance and submission. While these results provide no insight into a victim’s reasoning process, it may reflect the perceived effectiveness of adopting a cooperative behavioral style in these circumstances. For example, if attempting to control the offender may risk inviting a more extreme response in return, then cooperation may be effective in gaining compliance and lowering the risk of harm—without necessitating complete submission. This would also be in line with similar findings from Powers and Simpson (2012) and Fisher et al. (2007) who suggested that victims may select their response based on its parity with offender actions as well as the perceived effectiveness.

Prior studies have also highlighted the “leader–follower” nature of violent crime, with offenders being the primary aggressor and victims the “receiver” of that aggression (see Collins, 2008; van Dijk, 1994). This dynamic may explain the other non-hypothesized findings that offenders more commonly responded to victim dominance with hostility and victim cooperation with dominance. Being a non-probabilistic model, the IPC only models likely responses. In reality, a violent offender seeks to inflict some form of harm on the victim (Youngs et al., 2016), so their response will likely reflect this. They are unlikely to accept a victim’s attempt to exert control and thus escalate their aggression accordingly. This is supported by the frequent co-occurrence of offender dominant and hostile behaviors and the association between this behavioral style and victim hostility—suggesting that offenders may be more likely to escalate when victims express control or fight back then submit. Similarly, while for a victim behaving cooperatively may increase the likelihood of receiving cooperation from the offender in return, the association between this and offender dominance may indicate that even when they are complying, offenders are still intent on exerting their control.

In addition to these theoretical explanations, it is also possible that these unexpected findings arise from misclassifications between offender and victim behavior and the four styles of the IPC. As Bennell et al. (2001) noted, item misplacement is a common characteristic of interpersonal research (p. 163). The IPC’s bisecting structure means that individual behaviors will reflect elements of both axes—for example, dominance and cooperation or submission and hostility. Thus, while behaviors may be allocated to the style they most align with, this does not preclude their affiliation with the other axes. This is something that is difficult to control for and by using classifications drawn from prior, quantitative examinations of victim and offender behavior, the authors attempted to mitigate the extent this occurred. Given that the results primarily reflect the expected hypotheses and align with the principle of complementarity, it is unlikely that this occurred to an extent to skew the results in a significant way.

### Limitations

Three key limitations of this study and its current approach need to be acknowledged. First, the unequal distribution of crime types within the sample may affect the generalizability of the results. By including PA, SA, and DV, this research was able to provide unique and valuable insights into the victim–offender interaction across crime types. Future research could prioritize exploring whether the patterns remain consistent when greater numbers of SA and DV are included, or if crime subsamples are analyzed separately. Low numbers limited opportunities for this type of analysis and could have increased the likelihood of type two errors.

Second, the authors made multiple decisions during the parsing and categorization phases that affected how the sequences were analyzed. These included removing non-interactive behaviors, including a small number of the same victim–offender pairings across different incidents, condensing sequences of the same behavioral styles by the same actor into one action, and creating a “dominant/hostile” offender behavioral style. While these decisions were made to facilitate analysis, they are also aspects of interpersonal interactions themselves and reflect the complexity of this type of research. It is possible that including these behaviors and creating more complex behavior sequences would provide more nuanced insights into how victim, offender, and third parties respond to each other during violent crime. This was not possible in the current study, however, due to the large number of category combinations it would create compared to the number of cases/behaviors coded.

Third, this research focused exclusively on the victim–offender interaction and how they influence each other. However, it does not account for other influences such as those present in the environment or personal characteristics of either actor. These may also affect decision-making and behavioral style and are not considered here. This is a limitation of using behavioral sequence analysis as it does not as easily facilitate accounting for other potential variables as other methods such as crime script analysis. Future research could explore ways to incorporate other situational or personal variables into analysis to understand how these influence the victim–offender interaction and behavioral styles.

### Implications

The primary implication of these results is for the way we conceptualize the interaction between offenders and victims during violent crime. Far from one-sided or passive, these results show how, whether knowingly or unknowingly, victims have a direct and specific influence on offender behavior during their interaction. Following their application of the IPC to offender behavioral styles during sexual assault, Porter and Alison (2004) concluded that their result shows how different offender behaviors and abuse may create very different experiences for the victim. The present study indicates that the same may be said for the victim; specifically, how the victim acts and responds may result in a very different offender experience. To reiterate, this does not mean that the victim is responsible for what happens to them or the outcome of their experience, but failing to acknowledge the role of the victim limits our understanding of how violent crime occurs. Additionally, these results challenge the stereotype of the passive, helpless, ideal victim. It demonstrates that victims play an active, though unwilling, role in crime—influencing the offender through their behavior. Such results could be useful in raising awareness among investigators, victim services providers, and legal practitioners regarding the most common victim–offender behavioral sequences.

This study builds on the extensive body of work from Keatley and others that has used Behavior Sequence Analysis (BSA) to understand the nature and commission of violence, including sexual assault, homicide, and domestic violence. By using sequence analysis to map the interaction, these results demonstrate the value of applying this method to explore the victim’s role during crime and conceptualizations of victim agency. Further, these results extend those from prior research such as Bennell et al. (2001), Porter and Alison (2004), and Porter and Alison (2006). Importantly, by only examining the co-occurrence of offender and victim behaviors, these studies were only able to infer the impact of each actor on the others. The present study’s use of sequence analysis meant that it was able to empirically show the reciprocal influence and that the principle of complementarity applies to victim and offender behavior in both directions during physically assaultive crime. In particular, the unexpected findings, particularly as they relate to dominance, may indicate that victims are not simply taking their cue from, and responding in kind to, the offender. It is possible that the presence of dominance indicates that victims are not simply reacting to the offender as it reflects the expression of control. This requires some level of independence and autonomy—even if it is not successful.

However, the current research is limited in its ability to explain why a victim may engage in a particular behavioral style. While the IPC allows us to make inferences about an actor’s motivation—for example, behaving dominantly may reflect the desire to control another person or the situation while submission may reflect a desire to yield it—the extent to which this reflects real-life decision-making is unclear. These results suggest that victims may not simply be reacting and may possibly be attempting to exert influence over the situation and offender; however, this insight is only possible by asking victims directly. Thus, future research should aim to explore links between a victim’s behavioral style and characteristics such as offender behavior identified in this study and the situational and personal influences found in study two and any underlying reasoning or motivations. This will enable a true consideration of the victim as an active, though innocent, agent as opposed to passive participant during violent crime.
